# Understanding factors influencing safety and team functionality at operative vaginal birth through multidisciplinary perspectives: a mixed methods study

**DOI:** 10.1186/s12884-024-07075-w

**Published:** 2025-01-21

**Authors:** Sasha M. Skinner, Eleanor Kippen, Daniel L. Rolnik, Peter Neil, Ryan J. Hodges, Nadine Murry, Ben W. Mol, Arunaz Kumar

**Affiliations:** 1 Department of Obstetrics and Gynaecology, Women’s and Newborns, Monash Health, Clayton, VIC Australia; 2 Department of Obstetrics and Gynaecology, Monash University, Clayton, VIC Australia

**Keywords:** Operative vaginal birth, instrumental birth, communication, team functionality, human factors

## Abstract

**Background:**

Operative vaginal birth (OVB) relies on effective teamwork to optimise outcomes. This study aims to explore providers’ perspectives of factors influencing safety and team functionality at OVB.

**Methods:**

This mixed methods study involved four maternity sites at Monash Health, Australia. Surveys sent to healthcare providers invited quantitative and qualitative appraisal of safety and team functionality at OVB. Semi-structured interviews further explored themes emerging from survey responses. Categorical survey data were compared between staff roles using Pearson’s chi-squared tests. Thematic analysis of free-text survey responses and interviews identified themes influencing safety and team functionality at OVB.

**Findings:**

We received 100 survey responses from obstetric (*n* = 41), midwifery (*n* = 52) and paediatric (*n* = 7) staff, including senior (*n* = 49) and junior (*n* = 51) roles. Overall, 99% thought team communication should improve and 82% had witnessed practices outside protocol. Obstetric versus midwifery or paediatric staff were less likely to rate communication as low or very low quality (5% vs. 29%, *p* = 0.010). Reporting being extremely or very confident to escalate concerns was more likely in obstetric versus midwifery or paediatric staff (49% vs.12%, *p* = 0.003) and senior versus junior staff (44% vs. 10%, *p* = 0.008). Five overarching themes impacted on team functionality at OVB; (1) Quality of communication, (2) Preparation and risk assessment, (3) Leadership and interpersonal dynamics, (4) Transfer from Birth Unit to Operating Theatre, (5) Variation in clinical practice.

**Discussion:**

Some care providers perspectives of team functionality at OVB differed, with midwifery and paediatric staff more likely to report challenges with communication and were less empowered to escalate concerns. Training in factors impacting team functionality at OVB should be considered.

**Supplementary Information:**

The online version contains supplementary material available at 10.1186/s12884-024-07075-w.

## Background

Operative vaginal birth (OVB) accounted for 12% of all births in Australia 2021, and one in four births of first-time mothers [1]. OVB may be considered in the second stage of labour to expedite birth for maternal and/or fetal wellbeing, as an alternative to fully dilated caesarean section (FDCS). However, both OVB and FDCS are associated with increased risks of neonatal and maternal morbidity and mortality. In 2019, the Consultative Council on Obstetric and Paediatric Mortality and Morbidity (CCOPMM), a Victorian advisory body to the Minister of Health, reported that cases of poor outcomes resulting from OVB demonstrated similar themes of breakdown in team communication, loss of situational awareness, lack of escalation, and inadequate safety systems [2]. This report recommended a formal team time-out to improve situational awareness and decision-making in OVB, as well as ensuring staff were knowledgeable of current safety guidelines and avoided excessive traction to achieve vaginal birth. The “role and value of the whole healthcare team” alongside structured processes to assess and consider safety factors were emphasised.

The importance of human factors in healthcare to improve patient safety is increasingly recognised [3–5]. In maternity care, failure of human factors such as teamwork, communication and situational awareness, is responsible for over 70–80% of sentinel adverse events [6, 7]. Provision of safe and holistic intrapartum care relies on effective and respectful collaboration between members of the multidisciplinary team, as well as with the birthing woman and support person. In this high-acuity setting, decision-making is often time-critical and multifactorial, balancing the needs of mother and neonate, which may be conflicting. In this context, situational awareness involves the capacity to accurately identify, assess and anticipate multiple risks and events occurring in real-time, to therefore provide appropriate and timely care [7]. When performing technically complex births, such as OVB, clinicians can become task-focussed and thus susceptible to loss of situational awareness [8]. This may potentially lead to practices outside accepted safety guidelines, including excessive attempts at vaginal birth resulting in significant trauma to the mother and neonate, or limited capacity to anticipate and recognise complications [2, 9]. A team “time-out” refers to a pre-procedural safety checklist involving the healthcare team and patient which aims to introduce the team, summarise the planned procedure, identify any safety concerns, anticipate possible complications and outline escalation pathways. Safety checklists have been shown to improve team communication, adherence to safety guidelines and safety culture in many areas of medicine, including in maternity care [10–15]. Improving communication and collaboration of the healthcare team, the birthing woman and support person, has been shown to prevent adverse obstetric outcomes and improve maternal birth experience [16–18]. Enhancing team functionality at OVB is proposed to improve situational awareness, anticipation of potential complications, utilisation of available resources and adherence to safety guidelines [9, 19]. However, there is limited qualitative inquiry into the factors that impact teamwork and safety of OVB from healthcare workers’ perspectives.

## Methods

### Aim

This study aims to evaluate how team functionality and safety of OVB can be optimised through the perspectives of healthcare providers. In this study, team functionality refers to how healthcare staff perceive the dynamics, collaboration and performance of the multidisciplinary team present at an OVB, with particular attention to team communication, escalation, and adherence to safety guidelines, considering the 2019 CCOPMM recommendations.

### Study design

This study design is based on a pragmatist paradigm, in which multidimensional methods are encouraged to achieve the desired research objective whilst ensuring the research is contextually relevant to the subjects of its inquiry [20]. Pragmatism allows scope for the pluralist understanding of multiple truths, acknowledging that individuals can experience reality differently [21]. This is especially important when considering potential influences on multidisciplinary team functionality, where it is necessary to understand multiple perspectives and how they relate to each other.

We employed a mixed methods convergent sequential study design. First, to quantify the diversity of perspectives amongst a large cohort of staff involved in OVB, we utilised electronic surveys with both Likert-scale and free-text style questioning relating to OVB (Supplementary Material 1). The survey was developed by the researchers based on themes and recommendations relating to OVB in the 2019 CCOPMM report [2], in addition to exploring other factors identified by staff impacting safety of OVB. An interview guide (Supplementary Material 2) was then developed based on responses from the surveys and in collaboration with researchers of obstetric and midwifery clinical backgrounds. Semi-structured interviews were then performed to further explore emerging themes and nuances of contributory factors identified by staff.

### Team reflexivity

The diversity of our research team assists in maximizing data analysis credibility. SS, the primary author, is an obstetric trainee and concurrently completing a Doctor of Philosophy degree in obstetrics. EK is a junior midwife with four years’ experience in maternity care, and NM a senior midwife with more than 20 years’ experience. DR, RH, PN, BM and AK are specialist obstetricians with extensive experience in obstetrics academic and research design. This combination of junior and senior staff in various roles in maternity care enables comprehensive study design and interpretation from multiple perspectives within the clinical context.

### Setting

This study was performed at Monash Health encompassing four metropolitan public hospitals in Victoria, Australia, including one tertiary and three secondary sites. Monash Health is the largest maternity and perinatal provider in Victoria, providing care for over 10,000 births annually [22]. On review of medical records during the study period, the rates of forceps- and vacuum-assisted births at Monash Health were 10.5% and 4.8% of all term births, respectively. Obstetric staff must either be themselves credentialed or supervised by a credentialed practitioner to perform OVB. Midwives do not perform OVB, but do have an essential role in supporting the birthing woman as well as the obstetric provider, including preparing equipment and medications, receiving the neonate, managing complications and facilitating appropriate escalation as needed. The local protocol [23] indicates that all OVB are attended to by a paediatric staff member to care for the neonate. The local OVB protocol [23] gives recommendations regarding appropriate indications, prerequisites, precautions and contraindications, procedural limits, escalation pathways, complications and post birth cares for OVB with reference to international college guidelines. This protocol applies for all four maternity sites. Survey and interview data collection occurred from October to November 2021 at Monash Medical Centre, Dandenong and Casey Hospitals and then at Sandringham Hospital from March to May 2023 when this service joined the Monash Health network.

### Participants and recruitment

Participants were healthcare providers including junior and senior midwifery, obstetric and paediatric staff. Senior and junior midwifery staff were university-trained registered midwives with more than or less than five years of clinical experience, respectively. Senior obstetric and paediatric staff included medical specialists as well as senior obstetric trainees in the final two years of specialty training, as they would frequently supervise junior trainees performing OVB. Junior obstetric and paediatric staff included medical doctors in the first four years of training or not yet on the training program. Anonymous survey links were made available to all maternity care staff via staff e-mail and the maternity newsletter through convenience sampling. Across the Monash Health sites, this includes approximately 150 obstetric staff, 500 midwifery staff and 80 newborn staff. Following survey data collection and development of the interview guide, invitations to participate in interviews were similarly distributed to all maternity and perinatal staff via staff e-mail addresses. We utilised a mixture of convenience and purposive sampling for interviews, whereby recruitment continued until representation of senior and junior, obstetric and midwifery staff was achieved.

### Ethics

The study received ethical approval from the hospital Human Research Ethics Committee as a low-risk project. Participants gave informed and verbal consent to participate in the study. Survey responses were anonymous, and interview transcriptions were deidentified to maintain confidentiality.

### Data collection

Anonymous survey data were collected electronically from all four hospitals and completed independently by responders without assistance of research staff. Interviews were performed by authors SS and AK to further explore themes emerging from analysis of survey responses and involved five participants with representation of senior and junior midwifery and obstetric staff. Given surveys were anonymous, researchers were not aware of whether interview participants had responded to the survey. Participants were aware of the research aims prior to consenting to be interviewed. Interviews were conducted via video conferencing and audio recorded. The interview started by asking open-ended questions about participants’ roles at an OVB and factors they identified influencing team functionality and safety of OVB. As the interviews were semi-structured, the direction of the interview was guided by the participants, with a focus on exploratory enquiry. The interview guide (Supplementary Material 2) covered topics such as communication, teamwork, clinical practice in the context of recommended guidelines and escalation. The interview concluded by asking participants to share any suggestions they had to improve the physical or psychological safety of OVB for birthing women and their neonates. Interviews ranged from 20 to 28 min in duration. Interviews were transcribed verbatim, and transcriptions were checked for accuracy by one of the authors (SS) with the assistance Otter.ai software.

### Data analysis

Likert-scale survey responses were collated and compared between obstetric and midwifery or paediatric staff, as well as between junior and senior staff, using Pearson’s chi-squared test or fisher exact test for questions with less than five counts for any of the response options. The grouping of obstetric compared to midwifery and paediatric staff was chosen to reflect the perspectives of staff performing the OVB procedure (obstetric staff) compared to those present supporting the procedure and receiving the neonate (midwifery and paediatric staff). Statistical significance was defined as a p value < 0.05.

Deidentified interview transcriptions and free-text survey responses were thematically analysed per Braun and Clarke 6-step process [24, 25]. The data were read multiple times by authors SS, AK and EK, initially to gain familiarity and later to inductively and independently code the data, ensuring each transcript was reviewed by at least two researchers. In the first round of analysis, initial codes were agreed upon by the researchers and in subsequent rounds emerging themes were developed. Ongoing analysis and refinement of themes was performed to ensure themes applied to the entire dataset. Final themes and interpretations of the findings, in context of the research question, were agreed upon by the authors.

## Results

We received 100 responses to the survey. Of those, 41 were from obstetric, 52 midwifery, and seven from paediatric staff. There was an even distribution of responses from senior (*n* = 49) and junior staff (*n* = 51). The clinical roles of respondents are summarised in Table 1.

**Table 1:** Clinical roles of respondents

Midwifery or Paediatric staff	*n* = 59 (%)	Obstetric Staff	*n* = 41 (%)
Senior midwives	25 (42)	Specialist obstetrician	14 (34)
Junior midwives	27 (46)	Senior obstetric trainee	4 (10)
Specialist paediatrician	6 (10)	Junior obstetric trainee	23 (56)
Paediatric trainee	1 (2)		
**Junior Staff**	*n* = 51 (%)	**Senior Staff**	*n* = 49 (%)
Junior obstetric trainee	23 (45)	Specialist obstetrician	14 (29)
Paediatric trainee	1 (2)	Senior obstetric trainee	4 (8)
Junior midwives	27 (53)	Senior midwives	25 (51)
		Specialist paediatrician	6 (12)

*Data are presented as absolute numbers (n) and proportions (%).*

*Junior and senior midwives were defined as less than or greater than five years’ experience*,* respectively.*

*Senior obstetric trainees were those in the final two years of obstetric specialty training.*

Comparing responses from obstetric vs. midwifery and paediatric staff, obstetric staff were more likely to rate communication as high quality (56% vs. 29%, *p* = 0.010) and more likely to be very or extremely confident raising concern if they observed practices outside protocol (49% vs. 12%, *p* = 0.003). Compared to senior staff, junior staff were more likely to report never conducting or observing a team time-out prior to OVB (53% vs. 29%, *p* = 0.023) and less likely to be very or extremely confident raising concern if they observed practices outside protocol (10% vs. 45%, *p* = 0.008). Overall, 99% of healthcare staff thought communication at OVB could improve, with the majority (58%) reporting it should improve “a lot” or “a great deal”. Only 21% of staff reported that concerns would be raised by staff “usually” or “always” if practices outside protocol were observed. Likert-scale survey responses are summarised in Table 2. As there were relatively few responses from paediatric staff, we conducted a sensitivity analysis excluding paediatric staff responses with similar results (Supplementary Material 3).

**Table 2:** Perspectives of maternity and perinatal staff on team functionality at operative vaginal birth

	Overall (*n* = 100)	Midwifery or Paediatric staff (*n* = 59)	Obstetric staff (*n* = 41)	*p*-value	Junior staff (*n* = 51)	Senior staff (*n* = 49)	*p*-value
Currently, how effectively do you believe teams communicate during attempted OVB?							
Very high quality	2 (2)	2 (3)	0 (0)	**0.010**	0 (0)	2 (4)	0.289
High quality	40 (40)	17 (29)	23 (56)		20 (39)	20 (41)	
Neither high nor low quality	39 (39)	23 (39)	16 (39)		19 (37)	20 (41)	
Low quality	18 (18)	16 (27)	2 (5)		12 (24)	6 (12)	
Very low quality	1 (1)	1 (2)	0 (0)		0 (0)	1 (2)	
Do you think team communication at OVB could improve?							
A great deal	24 (24)	16 (27)	8 (20)	0.452	14 (28)	10 (20)	0.282
A lot	34 (34)	20 (34)	14 (34)		20 (39)	14 (29)	
A moderate amount	28 (28)	13 (22)	15 (37)		10 (20)	18 (37)	
A little	13 (13)	9 (15)	4 (10)		7 (14)	6 (12)	
None at all	1 (1)	1 (2)	0 (0)		0 (0)	1 (2)	
How often have you observed or conducted a team ‘time out’ prior to attempted OVB?							
Always	0 (0)	0 (0)	0 (0)	0.139	0 (0)	0 (0)	**0.023**
Usually	10 (10)	6 (10)	4 (10)		2 (4)	8 (16)	
Sometimes	20 (20)	8 (14)	12 (29)		11 (22)	9 (18)	
Rarely	29 (29)	16 (27)	13 (32)		11 (22)	18 (37)	
Never	41 (41)	29 (49)	12 (29)		27 (53)	14 (29)	
Have you witnessed OVB practices that are outside protocol?							
A great deal	3 (3)	2 (3)	1 (2)	0.513	2 (4)	1 (2)	**0.035**
A lot	6 (6)	5 (8.5)	1 (2)		1 (2)	5 (10)	
A moderate amount	28 (28)	19 (32)	9 (22)		21 (41)	7 (14)	
A little	45 (45)	25 (42)	20 (49)		18 (35)	27 (55)	
Not at all	11 (11)	5 (8.5)	6 (15)		5 (10)	6 (12)	
I am unsure of the Monash Health OVB protocol	7 (7)	3 (5)	4 (10)		4 (8)	3 (6)	
When you have observed OVB practices that are outside protocol, how often are concerns raised by other members of the team?							
Always	4 (4)	1 (2)	3 (7)	0.220	3 (6)	1 (2)	0.561
Usually	17 (17)	10 (17)	7 (17)		5 (10)	12 (25)	
Sometimes	35 (35)	23 (40)	12 (29)		20 (40)	15 (31)	
Rarely	24 (24)	17 (29)	7 (17)		13 (26)	11 (22)	
Never	4 (4)	1 (2)	3 (7)		2 (4)	2 (4)	
I am unsure of the Monash Health OVB protocol	9 (9)	4 (7)	5 (12)		4 (8)	5 (10)	
I have not observed OVB practices that are outside protocol	6 (6)	2 (3)	4 (10)		3 (6)	3 (6)	
*No response*	*1*	*1*	*0*		*1*	*0*	
When you observe OVB practices that are outside protocol, how comfortable do you feel about raising concern?							
Extremely confident	12 (12)	3 (5)	9 (22)	**0.003**	1 (2)	11 (22)	**0.008**
Very confident	15 (15)	4 (7)	11 (27)		4 (8)	11 (22)	
Somewhat confident	21 (21)	14 (24)	7 (17)		13 (26)	8 (16)	
Not so confident	34 (34)	24 (41)	10 (24)		20 (40)	14 (29)	
Not at all confident	9 (9)	8 (14)	1 (2)		6 (12)	3 (6)	
I am unsure of the Monash Health OVB protocol	6 (6)	3 (5)	3 (7)		4 (8)	2 (4)	
I have not observed OVB practices that are outside protocol	2 (2)	2 (3)	0 (0)		2 (4)	0 (0)	
*No response*	*1*	*1*	*0*		*1*	*0*	

Data are presented as absolute numbers (n) and proportions (%). p-values obtained using Chi-squared of Fisher’s Exact tests.

OVB = Operative Vaginal Birth

*NB. Eleven of the eighteen survey responders who reported being unsure of the protocol or having not observed OVB practices outside protocol*,* then answered one of the follow-up questions relating to raising concern when such practices were observed*,* which was inconsistent with their prior response.*

Qualitative analysis of free-text survey responses and semi-structured interviews identified five overarching themes impacting team functionality at OVB; (1) Quality of communication, (2) Preparation and risk assessment, (3) Leadership and interpersonal dynamics, (4) Transfer from Birth Unit to Operating Theatre and (5) Variation in clinical practice. These themes closely related to our research enquiry into communication, escalation and adherence to safety guidelines. Themes, subthemes and representative quotes are summarised in Table 3.

**Table 3:** Themes

Theme	Subtheme	Illustrative quotes
(1) Calibre of communication	Indirect communication	“We can sort of guess what might be happening, but it’s definitely not discussed with us…nobody actually tells you specifically, you just sort of overhear it.” (MW2, junior midwife) “Simply talk to one another and describe the degree of concern for the baby.” (PED4, specialist paediatrician)
	Unclear reasoning for decision-making	“I think a lot of women are confused and are unsure of what happened and why. And I think the midwives probably are too.” (MW1, senior midwife) “I don’t know necessarily why we choose to go down to theatre for a trial of forceps versus or forceps in the room” (MW2, junior midwife)
	Manner of communication impacting team performance	“This belittling and demanding communication can make junior staff, in particular, become frazzled and feel a great deal of pressure. This can lead to lack of ability to speak up or ask questions to put into documentation. It can also make the woman feel more concerned and uncomfortable.” (MW11, junior midwife) “Just more communication from O&G to midwifery, and not to get too upset if TXA [Tranexamic acid] and Augmentin [Amoxycillin-Clavulanic acid antibiotic] aren’t in the room straight away. It takes time and rapport to set up effectively for an instrumental by midwifery team and it can be so tough when communication isn’t effective.” (MW21, junior midwife) “I think they [the midwifery team] are just really good communicators… it’s a very open and very friendly environment.” (OB2, junior obstetric trainee)
	Inadequate consent	“I often feel women are inadequately counselled to make an informed decision” (MW42, senior midwife) “You might get a doctor sitting on the end of the bed talking to the woman saying, you know, you’ve been pushing for an hour, we haven’t seen much progress, we just need to “help your baby out” without actually necessarily saying what that is, or what that looks like, or the risks of that as well.” (MW2, junior midwife) “Communication to the woman is mostly inadequate.” (MW19, senior midwife)
(2) Preparation and risk assessment	Understanding capabilities of the team and role allocation	“It feels like no one really checks in… turns around and makes sure, Okay, who have we got in the room? We’ve got a really junior midwife. That’s it. Like, that’s probably not a good time to start the procedure.” (MW1, senior midwife) “Waiting until paediatrician arrives before delivery.” (MW17, junior midwife) “That would be my biggest suggestion…to make sure that all the right people are there. Explain what’s about to happen briefly. Make sure everyone knows that their roles are clearly defined. Make sure we have someone documenting, someone drawing up drugs, someone making sure all the equipment is present, making sure that the paediatrician was on the way or that some sort of person is there for the baby.” (MW1, senior midwife)
	Transparency of decision making and risk assessment	“More clear communication between obstetric and neonatal staff, particularly if there have been concerns about fetal wellbeing during labour” (PED1, specialist paediatrician) “I feel more confident when I’ve seen the doctor use that ultrasound prior to confirm position.” (MW1, senior midwife) “I don’t know, I haven’t had my hands inside the woman so I don’t know where the head is at …sometimes it can feel like decisions are made. And then if a woman asked you when you’re in recovery, why did that happen? I wouldn’t necessarily know the answer to that” (MW2, junior midwife)
	Pain relief	“I think there is often a lack of opportunity for any type of pain relief as well which can be super traumatic for the women…they often ask us afterwards why they had no pain relief!” (MW14, junior midiwfe) “I feel the only analgesia appropriate for trial of forceps is epidural/spinal to reduce the trauma women often describe.” (MW19, senior midwife)
(3) Leadership and interpersonal dynamics	Involvement of senior clinicians	“The seniority of the people in the room, that’s when I would be less confident, if particularly more junior doctors were the ones performing the instrumental.” (MW1, senior midwife) “Discuss with senior doctors prior to performing procedure and the consultant should provide a second opinion in Birth Suite and not book a trial of instrumental in Operating Theatre if procedure can be carried out on the ward.” (MW8, junior midwife) “They [the obstetric team] feel more comfortable, because if we go to Theatre, then they have a more senior person present.” (MW1, senior midwife)
	Interprofessional relationships and working with a familiar team	“I think it would depend on the situation for sure, and my relationship with a doctor. Like, some doctors, you know, you definitely feel much more comfortable with” (MW1, senior midwife) “I think maybe the hardest bit comes from because our doctors sort of cycle through and by the time we’re finally used to them, and what they do and how they communicate, they leave, and a new lot comes.” (MW2, junior midwife)
	Hierarchy and empowerment to escalate	“It doesn’t feel like you can necessarily say anything in that moment, particularly if it’s a consultant, obviously, doing the procedure, rather than like one of our registrars or something” (MW2, junior midwife) “I don’t feel like there’s a great level of maybe confidence of the midwives. I know I probably wouldn’t feel comfortable making those calls.” (MW1, senior midwife) “I feel least confident commenting to obstetric team as the paediatrician regarding number of pulls that should/shouldn’t be done.” (PED6, specialist paediatrician) “Everyone follows the hierarchy, they know when to escalate.” (OB1, specialist obstetrician)
(4) Transfer from Birth Unit to Operating Theatre	Change in team dynamics	“All healthcare workers in the theatre should provide a positive environment. As a midwife I often feel like an outsider which can make the experience awkward. We are all a team even if we are from different departments.” (MW5, junior midwife) “In Theatre… that’s obviously a very different experience, because Theatre is very in control of that space. But when they do happen upstairs [referring to Birth Suite], I think there’s enough people around, and I think we’ve got enough support.” (MW2, junior midwife)
	Difficulty communicating urgency	“But whenever we go down to Theatre for a trial of instrumental, it’s mainly the Theatre issue… it’s sometimes difficult to get things done quickly. It’s difficult to just get them on the same page…communication with the Theatre team is challenging” (OB1, specialist obstetrician) “The hardest part communication wise is getting down there and reinforcing the importance of why you need to be there…It makes it quite difficult for you to communicate how quickly you need to do something.” (OB2, junior obstetric trainee)
	Poor patient experience in theatre	“I think once you’re down in Theatre, there becomes this weird disconnect between the patient and what you’re doing… I think that change of venue sometimes messes with how clinicians might communicate with them.” (MW2, junior midwife) “A trial of forceps is a stressful and overwhelming experience for the woman and partner and midwife.” (MW19, senior midwife)
(5) Variation in clinical practice	Variation in clinician practice	“Sometimes there’s doctors that you know, this person is always going to go to Theatre, this person never goes to Theatre, which I don’t understand whether it’s just like a personal preference.” (MW1, senior midwife) “Each time is with a different consultant with a different method. It’s hard to get consistent practice. To be honest, it’s hard to apply the protocol because you just do what the consultant says.” (OB10, junior obstetric trainee) “There is a lot of variation in practice, for example with number of pulls with a vacuum delivery.” (PED7, specialist paediatrician) “There is also sometimes an inconsistency in where we do an OVB – Birth Suite or Operating Theatre. There needs to be a clearer policy as it appears to be based on the doctor’s experience as opposed to the clinical picture.” (MW41, senior midwife)
	Availability and clarity of guidelines and protocols	“I think it would definitely be worthwhile getting more information and education on the guidelines … so that we can identify if something hasn’t been done up to standard and can escalate that to whoever else. But yeah, I feel like I definitely don’t have that knowledge behind me.” (MW2, junior midwife) “I think everyone knows that there’s a certain amount of pulls or a certain amount of pop offs and all of those kinds of things. But I don’t think everyone knows exactly what that is.” (MW1, senior midwife) “Make it clearer and shared with paeds [the paediatric team] officially what actually one pull is defined as…Also how many pulls are allowed needs to be clearer as well.” (PED2, paediatric trainee) “I feel if we have a proper guideline, it would really be very helpful.” (OB1, specialist obstetrician)

*Themes and subthemes with examples of illustrative quotes from survey and interview data. Seniority and clinical role of staff providing quotes given in parenthesis.*

### Quality of communication

Healthcare providers described the importance of direct communication and clear reasoning for risk assessment and decision-making between the obstetric provider and supporting staff. Indirect communication led to significant confusion for both staff and patients.

*“We can sort of guess what might be happening*,* but it’s definitely not discussed with us…nobody actually tells you specifically*,* you just sort of overhear it.” (MW2*,* junior midwife)*.

The manner of communication was reported as an important factor that impacted team performance. Unclear or disrespectful communication was perceived to create a lack of understanding and/or disconnect between healthcare staff as well as with the patient, which impacted the dynamics of the team and potentially the safety of the procedure.

*“This belittling and demanding communication can make junior staff*,* in particular*,* become frazzled and feel a great deal of pressure. This can lead to lack of ability to speak up or ask questions to put into documentation. It can also make the woman feel more concerned and uncomfortable.” (MW11*,* junior midwife)*.

Communication with birthing woman was repeatedly identified as an important aspect of optimal care. Concern was raised about challenges in obtaining adequate consent for the procedure in an emergency setting or using language that “downplayed” significant risks.

*“I often feel women are inadequately counselled to make an informed decision” (MW42*,* senior midwife)*.

Conversely, obstetric staff were more likely to perceive the current quality of communication at OVB as effective, as reflected in quantitative analysis.

*“I think they [the midwifery team] are just really good communicators… it’s a very open and very friendly environment.” (OB2*,* junior obstetric trainee)*.

### Preparation and risk assessment

The next theme, related to both communication and escalation, was the need to adequately prepare for the birth, including being aware of the capabilities and limitations of the team, accurately assessing risks and anticipating potential complications. Participants spoke about appropriate allocation of roles and ensuring all necessary members of the team were present for the birth, in particular awaiting arrival of someone adequately skilled to care for the neonate. This process was reliant on multidisciplinary communication.

*“It feels like no one really checks in… turns around and makes sure*,* Okay*,* who have we got in the room? We’ve got a really junior midwife. That’s it. Like*,* that’s probably not a good time to start the procedure.” (MW1*,* senior midwife)*.

*“More clear communication between obstetric and neonatal staff*,* particularly if there have been concerns about fetal wellbeing during labour” (PED1*,* specialist paediatrician)*.

Use of ultrasound to confirm fetal head position was noted to increase objectivity of risk assessment, which increased the confidence of staff.

*“I feel more confident when I’ve seen the doctor use that ultrasound prior to confirm position.” (MW1*,* senior midwife)*.

Ensuring adequate pain relief was also highlighted as an important aspect of preparation for an OVB.

*“I think there is often a lack of opportunity for any type of pain relief as well which can be super traumatic for the women…they often ask us afterwards why they had no pain relief!” (MW14*,* junior midwife)*.

### Leadership and interpersonal dynamics

A key theme related to escalation in the context of team functionality and safety, was the role of leadership and interpersonal dynamics of the team. Higher seniority of staff present at the birth was noted to improve confidence of the team, ensure appropriate decision-making and readily available support in case of complication.

*“The seniority of the people in the room*,* that’s when I would be less confident*,* if particularly more junior doctors were the ones performing the instrumental.” (MW1*,* senior midwife)*.

On the other hand, higher seniority of involved staff and the team’s hierarchical structure were identified as barriers impacting confidence to escalate. Midwifery and paediatric staff interviewed expressed that they would not feel comfortable escalating if they had concerns about the birth.

*“It doesn’t feel like you can necessarily say anything in that moment*,* particularly if it’s a consultant*,* obviously*,* doing the procedure*,* rather than like one of our registrars or something” (MW2*,* junior midwife)*.

Conversely, senior obstetric staff reported feeling more comfortable with existing escalation pathways.

*“Everyone follows the hierarchy*,* they know when to escalate.” (OB1*,* specialist obstetrician)*.

The familiarity of the team was also a key factor reported to impact staff confidence and comfort to escalate concerns, with challenges noted with constant rotation of staff through the service.

*“I think maybe the hardest bit comes from because our doctors sort of cycle through and by the time we’re finally used to them*,* and what they do and how they communicate*,* they leave*,* and a new lot comes.” (MW2*,* junior midwife)*.

### Transfer from birth unit to operating theatre

The Operating Theatre environment was noted to be a particularly challenging setting to optimise team functioning, particularly regarding communication and escalation. The sudden change in the team was acknowledged as disruptive to interdisciplinary communication and team cohesion.

*“All healthcare workers in the theatre should provide a positive environment. As a midwife, I often feel like an outsider which can make the experience awkward. We are all a team even if we are from different departments.” (MW5*,* junior midwife)*.

Obstetric staff noted that it was challenging to escalate and communicate their concerns about the urgency of the birth with theatre staff.

“But whenever we go down to Theatre for a trial of instrumental… it’s sometimes difficult to get things done quickly. It’s difficult to just get them on the same page…communication with the Theatre team is challenging” (OB1, specialist obstetrician).

Conversely, some staff felt more comfortable in the Operating Theatre, as this meant there was more senior obstetric support available.

*“They [the obstetric team] feel more comfortable*,* because if we go to Theatre*,* then they have a more senior person present.” (MW1*,* senior midwife)*.

Healthcare staff reported births in the Operating Theatre to be particularly stressful for the birthing woman and support person. This more medicalised environment could lead to a greater disconnect between the birthing woman and the healthcare team, with less focus on communication and rapport in the context of increased clinical acuity.

*“I think once you’re down in Theatre*,* there becomes this weird disconnect between the patient and what you’re doing… I think that change of venue sometimes messes with how clinicians might communicate with them.” (MW2*,* junior midwife)*.

### Variation in clinical practice

The final overarching theme was most strongly related to our exploration of adherence to accepted safety guidelines. Healthcare staff noted variation in clinical practice, particularly regarding the indication for transferring to the operating theatre, the methods of expediting birth and the number of tractions acceptable to perform. This was noted to depend on the clinician attending the birth, the location of the birth and clinical context. Such variation made the reasoning of decision-making unclear and unpredictable, potentially limiting their capacity to apply protocol or optimally support the obstetric provider.

*“Each time is with a different consultant with a different method. It’s hard to get in consistent practice. To be honest*,* it’s hard to apply the protocol because you just do what the consultant says.” (OB10*,* junior obstetric trainee)*.

Guidelines and policies were seen as useful tools to assist this and improve safety of OVB, but were thought to be currently lacking or not accessible. Healthcare staff sought education and transparency around accepted safety guidelines and shared terminology, such what constitutes one “pull”.

*“I think it would definitely be worthwhile getting more information and education on the guidelines … so that we can identify if something hasn’t been done up to standard and can escalate that to whoever else. But yeah*,* I feel like I definitely don’t have that knowledge behind me.” (MW2*,* junior midwife)*.

*“Make it clearer and shared with paeds [referring to the paediatric team] officially what actually one pull is defined as…Also how many pulls are allowed needs to be clearer as well.” (PED2*,* paediatric trainee)*.

## Discussion

This study provides insight into the perspectives of healthcare staff on the factors impacting safety and team functionality at OVB. Prominent themes included the quality of communication, preparation and risk assessment, leadership and interpersonal dynamics, transfer from Birth Unit to the Operating Theatre and variation in clinical practice. The themes identified had clear interplay with our study enquiry into factors of communication, escalation and adherence to safety guidelines. Variation in perspectives of different members of the healthcare team were congruent in the quantitative and qualitative analysis, particularly regarding quality of communication and capacity to escalate.

All healthcare providers acknowledged the importance of optimising communication at OVB, both within the healthcare team and with the birthing woman. However, concerns with communication were more likely to be highlighted by midwifery and paediatric staff. In particular, staff reported that inadequate communication between the obstetric provider and other attending staff limited awareness of clinical risk assessment, reasoning behind decision-making or required roles and expectations, leading to confusion and inability to optimally support the obstetric provider or provide an explanation to the birthing woman. Communicating the needs and limitations of the team is particularly important in maternity care, where teams are dynamic with frequently rotating rosters, and thus involve members with variable experiences and capabilities [9]. The manner of communication was also perceived as important in getting optimal team performance and outcomes. This is supported by a recent retrospective cohort study [16] which found that interprofessional communication training could significantly reduce adverse obstetric events.

Concerns were also expressed regarding communication with the birthing woman, a fundamental member of the team at an OVB. In particular, responders reflected on the challenges in obtaining adequate consent from a birthing woman in an emergency situation, such as OVB. This is in keeping with previous studies [26], which highlight factors impacting intrapartum consent, such as pain and the emergency nature of decision-making. Dedicated efforts to involve birthing women in decision-making and supporting their autonomy was recognised by staff as a crucial influence on psychological safety. Previous studies [27, 28] have demonstrated increased risk of negative birth experience and development of post-traumatic stress disorder with emergency operative birth, including OVB. However, a recent qualitative analysis of women who had an OVB, identified that being part of the team and feeling empowered were key to facilitating a positive birth experience [18]. 

Confidence of staff and perceived functioning of the team at OVB were related to leadership and interpersonal relationships. Greater seniority of the obstetric provider assisting the birth improved perceived team confidence and safety, but was also a significant barrier to staff feeling empowered to escalate concerns. Additionally, poorly developed relationships within the team and lack of awareness of existing best practice guidelines reduced staff confidence to escalate. Midwifery and paediatric or junior staff were less likely to feel empowered to escalate, compared to obstetric and senior staff. These contrasting perspectives likely reflect differences in power dynamics associated with the traditional hierarchical structure of the team. A recent study [6] acknowledged that care is more effective and coordinated, particularly in a crisis situation, in teams where leaders “flatten the hierarchy to promote information exchange”.

Many responders acknowledged additional challenges in communication, escalation and team cohesion at OVB performed in the Operating Theatre. Some staff felt like “outsiders” or found it challenging to be “on the same page” in the Operating Theatre, reflecting a deterioration in interprofessional relations and team dynamics. The introduction of new team members, change in environment and loss of additional midwifery support contributed to this fragmentation. The change in location was noted to be stressful and overwhelming for both staff and patients, with one staff member describing greater “disconnect” with the patient in the more medicalised environment. It was acknowledged that births in the Operating Theatre were typically more high-risk which contributed to stress of the team. Conversely, some staff reported that the additional senior obstetric support present at births in the Operating Theatre was reassuring and improved confidence.

Variations in clinical practice contributed further to uncertainty amongst the team, particularly relating to risk assessment or reasoning for management. Proposed factors influencing variation in practice included clinician preferences and skill, differences in hospital policy, location of the attempted OVB and the clinical context of the birth. This theme was strongly related to adherence to safety guidelines, with staff universally advocating for the development of clear and accessible guidelines and policies as well as shared understanding of terminology, as these were recognised as useful tools to support consistency of safe practice.

An important limitation of this study is that it was performed at one healthcare network, which may limit generalisability. However, it did include staff across multiple hospital campuses with differing levels of clinical acuity. As data were collected from voluntary surveys and interviews, we captured only a subset of the staff involved in OVB, which introduces risk of selection bias. Whilst this study obtained relatively balanced representation of senior and junior staff, as well as of staff performing and supporting OVB, there were relatively few paediatric staff who participated in the study, increasing the risk of bias and reducing generalisability of those responses. Authors SS and AK who conducted the interviews are obstetric care providers, which may have helped build rapport, but may also have influenced interviewee responses. Whilst this study focussed on healthcare staff perceptions of team functionality and safety of OVB, it is crucial that future research include the perspectives of birthing women.

This study highlights the importance of non-technical skills to support team functionality and safety of OVB, in keeping with previous studies [29, 30] identifying the crucial role of non-technical skills in OVB competency. Healthcare staff in this study called for increased clarity of multidisciplinary communication and transparency in risk assessment, prioritising involving and engaging the birthing women, fostering positive interdisciplinary relationships especially in the context of OVB in the Operating Theatre and greater access to safety guidelines. Further research should explore methods to improve non-technical skills, such as those identified in this study, and assess the potential to improve the physical and psychological safety of OVB. Our findings suggest that this is likely to require a multimodal approach with incorporation of education, skills training, guideline accessibility, systems changes and cultural transformation.

## Conclusion

Healthcare providers identified multiple factors that they perceived to impact team functionality and patient safety at OVB. Perspectives on communication and empowerment to escalate differed between members of the team. Acknowledging the perspectives of all staff members and how they may differ is a crucial first step to understand key factors to target in order to comprehensively improve team functionality and safety at OVB. Improved training in non-technical skills, alongside procedural technique, should be considered to ensure and psychological safety through excellence in team-based care.

## Electronic Supplementary Material

Below is the link to the electronic supplementary material.

Supplementary Material 1

Supplementary Material 2

Supplementary Material 3

## Data Availability

The data used and analysed during the current study are available from the corresponding author on reasonable request.

## List of abbreviations

CCOPMM: Consultative Council on Obstetric and Paediatric Mortality and Morbidity
FDCS: Fully dilated caesarean section
OVB: Operative vaginal birth
