# Supplementary material for: Understanding factors influencing safety and team functionality at operative vaginal birth through multidisciplinary perspectives: a mixed methods study
Source: BMC Pregnancy Childbirth. 2025 Jan 21;25:47. doi: 10.1186/s12884-024-07075-w (PMC11753089; doi:10.1186/s12884-024-07075-w)
Supplement: Supplementary file 1 — Supplementary Material 1 [file 12884_2024_7075_MOESM1_ESM.docx]

**Supplementary Material 1. Survey Questions**

What is your role at Monash Health?

Currently, how effectively do you believe teams communicate during attempted OVB?

Do you think team communication at OVB could improve?

How often have you observed or conducted a team 'time out' prior to attempted OVB?

Have you witnessed OVB practices that are outside recommended guidelines?

(e.g. number of pulls, analgesia, escalation)

When you have observed OVB practices that are outside recommended guidelines, how often are concerns raised by other members of the team?

When you observe OVB practices that are outside recommended guidelines, how comfortable do you feel about raising concern?

Please describe which aspects of an OVB you are least confident or comfortable with.

(Please consider aspects that are relevant to your role at an OVB)

Please describe any other suggestions you have to improve OVB outcomes.
