# Supplementary material for: Understanding factors influencing safety and team functionality at operative vaginal birth through multidisciplinary perspectives: a mixed methods study
Source: BMC Pregnancy Childbirth. 2025 Jan 21;25:47. doi: 10.1186/s12884-024-07075-w (PMC11753089; doi:10.1186/s12884-024-07075-w)
Supplement: Supplementary file 2 — Supplementary Material 2 [file 12884_2024_7075_MOESM2_ESM.docx]

**Supplementary Material 2. Interview Guide**

Please describe your role(s) during an operative vaginal birth

How comfortable or confident do you feel being involved in an OVB. Please explain why.

What aspects of being involved in an OVB are you least comfortable or confident with. Please explain why.

From your perspective, how effective is communication during attempted OVB? This may include communication between members of the clinical team as well as with birthing women. Please explain why.

How effectively do you believe care providers work as a team during attempted OVB? Please explain why.

Are you aware of recommended guidelines for performing OVB safely (e.g., adhering to maximum number of pulls, appropriate analgesia, timely escalation)

What is your impression of current OVB practices in relation to recommended guidelines?

If necessary, how comfortable do you feel voicing concerns or escalating during an OVB? Please explain why and how you would typically approach this situation.

How often is ultrasound used prior to OVB to assess fetal head position. Please explain why you think this is.

What clinical and/or professional skills or knowledge of care providers do you think would help improve the physical and psychological safety of OVB for birthing mothers.

Please describe any other suggestions you have to improve OVB outcomes.
