# Supplementary material for: Understanding factors influencing safety and team functionality at operative vaginal birth through multidisciplinary perspectives: a mixed methods study
Source: BMC Pregnancy Childbirth. 2025 Jan 21;25:47. doi: 10.1186/s12884-024-07075-w (PMC11753089; doi:10.1186/s12884-024-07075-w)
Supplement: Supplementary file 3 — Supplementary Material 3 [file 12884_2024_7075_MOESM3_ESM.docx]

**Supplementary Material 3. Sensitivity Analysis Excluding Responses from Paediatric staff**

|  | **Overall**  (n = 93) | **Midwifery staff**  (n = 52) | **Obstetric staff** (n = 41) | p-value | **Junior staff**  (n = 50) | **Senior staff**  (n = 43) | p-value |
| --- | --- | --- | --- | --- | --- | --- | --- |
| Currently, how effectively do you believe teams communicate during attempted OVB? | | | | | | | |
| Very high quality | 2 (2) | 2 (4) | 0 (0) | **0.003** | 0 (0) | 2 (4) | 0.415 |
| High quality | 39 (42) | 16 (31) | 23 (56) |  | 20 (40) | 19 (41) |  |
| Neither high nor low quality | 35 (38) | 19 (37) | 16 (39) |  | 19 (38) | 16 (37) |  |
| Low quality | 17 (18) | 15 (29) | 2 (5) |  | 11 (22) | 6 (14) |  |
| Very low quality | 0 (0) | 0 (0) | 0 (0) |  | 0 (0) | 0 (0) |  |
| Do you think team communication at OVB could improve? | | | | | | | |
| A great deal | 23 (25) | 15 (29) | 8 (20) | 0.199 | 14 (28) | 9 (21) | 0.489 |
| A lot | 32 (34) | 18 (35) | 14 (34) |  | 19 (38) | 13 (30) |  |
| A moderate amount | 24 (26) | 9 (17) | 15 (37) |  | 10 (20) | 14 (33) |  |
| A little | 13 (14) | 9 (17) | 4 (10) |  | 7 (14) | 6 (14) |  |
| None at all | 1 (1) | 1 (2) | 0 (0) |  | 0 (0) | 1 (2) |  |
| How often have you observed or conducted a team 'time out' prior to attempted OVB? | | | | | | | |
| Always | 0 (0) | 0 (0) | 0 (0) | 0.133 | 0 (0) | 0 (0) | **0.025** |
| Usually | 10 (11) | 6 (12) | 4 (10) |  | 2 (4) | 8 (19) |  |
| Sometimes | 19 (20) | 7 (13) | 12 (29) |  | 11 (22) | 8 (19) |  |
| Rarely | 26 (28) | 13 (25) | 13 (32) |  | 11 (22) | 15 (35) |  |
| Never | 38 (41) | 26 (50) | 12 (29) |  | 26 (52) | 12 (28) |  |
| Have you witnessed OVB practices that are outside protocol? | | | | | | | |
| A great deal | 3 (3) | 2 (4) | 1 (2) | 0.105 | 2 (4) | 1 (2) | **0.014** |
| A lot | 6 (6) | 5 (10) | 1 (2) |  | 1 (2) | 5 (12) |  |
| A moderate amount | 27 (29) | 18 (35) | 9 (22) |  | 21 (42) | 6 (14) |  |
| A little | 42 (45) | 22 (42) | 20 (49) |  | 18 (36) | 24 (56) |  |
| Not at all | 11 (12) | 5 (10) | 6 (15) |  | 5 (10) | 6 (14) |  |
| I am unsure of the Monash Health OVB protocol | 4 (4) | 0 (0) | 4 (10) |  | 3 (6) | 1 (2) |  |
| When you have observed OVB practices that are outside protocol, how often are concerns raised by other members of the team? | | | | | | | |
| Always | 4 (4) | 1 (2) | 3 (7) | **0.033** | 3 (6) | 1 (2) | 0.480 |
| Usually | 17 (18) | 10 (20) | 7 (17) |  | 5 (10) | 12 (28) |  |
| Sometimes | 34 (37) | 22 (43) | 12 (29) |  | 20 (41) | 14 (33) |  |
| Rarely | 22 (24) | 15 (29) | 7 (17) |  | 13 (27) | 9 (21) |  |
| Never | 4 (4) | 1 (2) | 3 (7) |  | 2 (4) | 2 (5) |  |
| I am unsure of the Monash Health OVB protocol | 5 (5) | 0 (0) | 5 (12) |  | 3 (6) | 2 (5) |  |
| I have not observed OVB practices that are outside protocol | 6 (6) | 2 (4) | 4 (10) |  | 3 (6) | 3 (7) |  |
| When you observe OVB practices that are outside protocol, how comfortable do you feel about raising concern? | | | | | | | |
| Extremely confident | 12 (13) | 3 (6) | 9 (22) | **0.001** | 1 (2) | 11 (26) | **<0.001** |
| Very confident | 15 (16) | 4 (8) | 11 (27) |  | 4 (8) | 11 (26) |  |
| Somewhat confident | 21 (23) | 14 (27) | 7 (17) |  | 13 (27) | 8 (19) |  |
| Not so confident | 32 (34) | 22 (43) | 10 (24) |  | 20 (41) | 12 (28) |  |
| Not at all confident | 7 (7) | 6 (12) | 1 (2) |  | 6 (12) | 1 (2) |  |
| I am unsure of the Monash Health OVB protocol | 3 (3) | 0 (0) | 3 (7) |  | 3 (6) | 0 (0) |  |
| I have not observed OVB practices that are outside protocol | 2 (2) | 2 (4) | 0 (0) |  | 2 (4) | 0 (0) |  |
| *Data are presented as absolute numbers (n) and proportions (%). p-values obtained using Chi-squared of Fisher’s Exact tests.*  *OVB = Operative Vaginal Birth* | | | | | | | |
